# Coordinated Response to Imported Vaccine-Derived Poliovirus Infection, Barcelona, Spain, 2019–2020

**DOI:** 10.3201/eid2705.204675

**Published:** 2021-05

**Authors:** Dolores Álamo-Junquera, Julieta Politi, Pere Simón, Romina Dieli-Crimi, Ricardo Pujol Borrell, Roger Colobran, Monica Martínez-Gallo, Magda Campins, Andrés Antón, Juliana Esperalba, Cristina Andrés, María Gema Codina, Eva Polverino, M. Rosa Narciso, Emilia Molinero, Cristina Rius

**Affiliations:** Public Health Agency of Barcelona, Barcelona, Spain. (D. Álamo-Junquera, J. Politi, P. Simón, E. Molinero, C. Rius);; Parc de Salut Mar, Barcelona (J. Politi);; Hospital Universitari Vall d'Hebron, Barcelona (R. Dieli-Crimi, R. Pujol Borrel, R. Colobran, M. Martínez-Gallo, M. Campins, A. Antón, J. Esperelba, M. Gema Codina, E. Polverino);; Universitat Autònoma de Barcelona, Barcelona (R. Colobran, M. Martínez-Gallo, M. Campins, A. Antón, J. Esperelba, M. Gema Codina);; Institut Català de la Salut, Barcelona (M.R. Narciso);; Centro de Investigación Biomédica en Red de Epidemiología y Salud Pública, Barcelona (C. Rius)

**Keywords:** vaccine-preventable diseases, surveillance, poliovirus vaccine, oral, adverse effects, immunologic deficiency syndromes, complications, disease eradication, viruses, Barcelona, Spain, vaccines

## Abstract

In 2019, the Public Health Agency of Barcelona, Spain, was notified of a vaccine-derived poliovirus infection. The patient had an underlying common variable immunodeficiency and no signs of acute flaccid paralysis. We describe the ongoing coordinated response to contain the infection, which included compassionate-use treatment with pocapavir.

In the nearly 30 years since the inception of the Global Polio Eradication Initiative (GPEI) by the World Health Organization (WHO), polio eradication efforts have decreased the number of wild poliovirus (WPV) cases by more than 99%, from 350,000 worldwide in 1988 to only 143 reported cases in 2019 (*1*). The trivalent Sabin vaccine (oral polio vaccine [OPV]), chosen for the eradication program, results in a temporary intestinal infection in immunocompetent persons. These vaccine-derived strains can revert to being neurovirulent after vaccination, especially in immunocompromised patients, leading to vaccine-associated paralytic poliomyelitis or chronic poliovirus infection (*2*).

The Polio Eradication and Endgame Strategic Plan 2013–2018 established by the GPEI proposed introducing inactivated polio vaccine (IPV) into routine childhood vaccination and eventually removing OPV from global use (*3*). This transition began in 2016 with the replacement of trivalent OPV with bivalent OPV types 1 and 3 (*4*).

After WHO declared a Public Health Emergency of International Concern regarding WPV in 2014 (*5*), worldwide eradication of indigenous WPV serotype 2 was declared in 2015 (*6*). Worldwide eradication of WPV serotype 3 was achieved in 2019, after the last case of WPV serotype 3 was reported in Nigeria in 2012 (*7*). Nonetheless, endemic transmission of WPV serotype 1 continues to cause cases in Afghanistan and Pakistan (*8*).

In Spain, the last endemic case of WPV occurred in 1988, which prompted improvement in polio vaccination coverage and replacement of OPV with IPV in 2004. Although the WHO’s European Region has been certified polio-free since 2002, the risk for imported cases of WPV or vaccine-derived polioviruses (VDPVs) from other countries still exists.

Primary immunodeficiencies are a heterogeneous group of disorders with a substantial hereditary component. In patients with primary immunodeficiencies, the immune response to microbial pathogens is defective, leading to higher susceptibility to infections, which can then become chronic (*9*). These patients can become infected by immunization if they receive live vaccines (*10*), and these infections can pose a risk to immunodeficient contacts and potentially jeopardize the success of the GPEI (*11*).

## Case Report

In May 2019, the Public Health Agency of Barcelona (PHAB) was notified about a poliovirus infection in an asymptomatic person with a primary immunodeficiency, identified through ongoing nonpolio enterovirus surveillance at Vall d’Hebron Hospital (Barcelona), which conducted VP1 sequencing of all detected enteroviruses (Figure).

**Figure:**
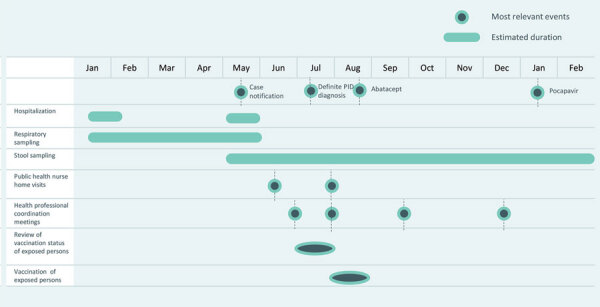
Timeline of public health actions in response to an imported vaccine-derived poliovirus infection, Barcelona, Spain, 2019–2020. PID, primary immunodeficiency.

The patient, a 26-year-old man, tested positive for enterovirus (identified as poliovirus types 1 and 3) in a pharyngeal swab specimen. His medical history included a common variable immunodeficiency that had been diagnosed in 2012 and treated with intravenous immunoglobulin-replacement therapy.

Born in Pakistan, the patient had lived in Barcelona since 2010, and the household included 4 family members (parents, partner, and child). He was a cook at a local restaurant. The patient was adequately vaccinated with 3 polio vaccine doses in 1993 in Pakistan (probably trivalent OPV) and 1 dose of IPV in 2015 in Barcelona. His child was correctly vaccinated with IPV, in accordance with the vaccine schedule of Catalunya, of which Barcelona is the capital. His parents reported previous vaccination with 3 OPV doses in Pakistan. His partner recalled receiving their most recent OPV dose in February 2017 before leaving Pakistan for Barcelona; they had also received 3 doses of OPV as a child in Pakistan.

Neither the patient nor his 4 household members reported recent travel to polio-endemic areas. However, they recalled receiving visits by relatives and friends who frequently traveled to Pakistan.

After the notification, a public health nurse visited the patient and his family to collect epidemiologic information and stool samples and to explain how to prevent transmission. National public health authorities also were notified.

The patient’s stool samples were positive for poliovirus types 1 and 3. The grade of divergence to the parent Sabin strain (2.7% for type 1 and 1.5% for type 3) was consistent with a 2-year reproduction time. A recovered respiratory sample collected from the patient in January 2019 also tested positive for both strains. In contrast, the stool samples of the 4 household members tested negative for poliovirus.

To coordinate the epidemiologic investigation and determine future actions, PHAB convened all the healthcare professionals involved in the patient’s follow-up, including primary-care clinicians and hospital specialists. Polio immunization coverage in the patient’s surrounding community was confirmed to be >95%, and environmental samples did not show any evidence of circulation in the community. Contact tracing, beyond family members, included work contacts, healthcare personnel, and other patients. A total of 59 persons exposed were vaccinated with IPV. In addition, a contact-precautions alert was added to the patient’s electronic medical record. Because he worked as a food handler, and given the possibility of a long excretion period, PHAB authorized a medical leave and suggested a reorientation of his professional career.

Further testing of the patient was indicated after the first meeting. Massive parallel sequencing identified a mutation supporting the diagnosis of CTLA-4 deficiency, a dominant monogenic disease, specifically an autoimmune lymphoproliferative syndrome, type V, which is treatable. Abatacept (a recombinant soluble form of CTLA-4) was started in August 2019 and resulted in substantial clinical improvement. Specific treatment with pocapavir (authorized for compassionate use) was provided by ViroDefense Inc. (Chevy Chase, MD, USA) and was started in January 2020.

To date, the patient’s poliovirus excretion is monitored by monthly stool samples and pharyngeal swab specimens every 6 months. Although the most recent pharyngeal swab specimens was negative, stool samples remain intermittently positive. Stool samples provided by household members every 6 months continue to be negative. No onward transmission has been identified.

## Conclusions

We report a chronic vaccine-derived poliovirus excretion in a person with a primary immunodeficiency who was living in a polio-free country. Immunodeficient patients have a high risk for becoming chronic primary immunodeficiency carriers and face an increased risk for VDPV complications. In a nonendemic area, the most likely infection source is secondary exposure to vaccine-related strains imported by persons vaccinated in countries that have ongoing vaccination with OPV.

Because patients with antibody deficiency are susceptible to vaccine-associated paralytic poliomyelitis, OPV and other live vaccines are contraindicated in such persons. However, the diagnosis of an antibody deficiency often is delayed, making this recommendation challenging. 

The grade of divergence to the parent Sabin strain is estimated to occur at a rate of 1.1% per year for the capsid protein (VP1) region, which for this case is consistent with a 2-year excretion. This finding, along with the OPV vaccination of the patient’s partner in 2017, supports secondary exposure to be the most likely source of infection.

In a polio-free country, the occurrence of vaccine-derived carriers is infrequent. However, persons with immunodeficiency-related VDPV infection can maintain prolonged excretions while remaining asymptomatic (*12*). Detection of asymptomatic poliovirus infections entails difficulties, as in the case we have described. Physicians treating patients with primary immunodeficiency might encourage their screening by including serial stool samples for all newly identified patients with primary immunodeficiency. Results from stool specimen screening should be included in a global poliovirus surveillance reporting system (*13*).

Antivirals represent a potential means to manage immunodeficiency-related VDPV excretors and the risk their conditions represent to eradication efforts (*14*). Currently, pocapavir is being considered for use in poliovirus-excreting patients with primary immunodeficiency on a compassionate-use basis (*15*). The case-patient we describe has been treated with pocapavir, although viral excretion remained active at the time of this report. Further research is needed to identify effective antiviral drugs. In conclusion, in certified polio-free countries, clinical and virologic surveillance guidelines must address asymptomatic poliovirus carriers and the need for screening in immunocompromised persons.
